# Interpersonal sensorimotor communication shapes intrapersonal coordination in a musical ensemble

**DOI:** 10.3389/fnhum.2022.899676

**Published:** 2022-09-29

**Authors:** Julien Laroche, Alice Tomassini, Gualtiero Volpe, Antonio Camurri, Luciano Fadiga, Alessandro D’Ausilio

**Affiliations:** ^1^Center for Translational Neurophysiology of Speech and Communication, Italian Institute of Technology, Ferrara, Italy; ^2^Casa Paganini – InfoMus Research Centre, Department of Informatics, Bioengineering, Robotics and Systems Engineering (DIBRIS), University of Genova, Genova, Italy; ^3^Sezione di Fisiologia, Dipartimento di Neuroscienze e Riabilitazione, Università di Ferrara, Ferrara, Italy

**Keywords:** interpersonal coordination, intrapersonal coordination, embodied music cognition, music ensemble performance, multiple timescale

## Abstract

Social behaviors rely on the coordination of multiple effectors within one’s own body as well as between the interacting bodies. However, little is known about how coupling at the interpersonal level impacts coordination among body parts at the intrapersonal level, especially in ecological, complex, situations. Here, we perturbed interpersonal sensorimotor communication in violin players of an orchestra and investigated how this impacted musicians’ intrapersonal movements coordination. More precisely, first section violinists were asked to turn their back to the conductor and to face the second section of violinists, who still faced the conductor. Motion capture of head and bow kinematics showed that altering the usual interpersonal coupling scheme increased intrapersonal coordination. Our perturbation also induced smaller yet more complex head movements, which spanned multiple, faster timescales that closely matched the metrical levels of the musical score. Importantly, perturbation differentially increased intrapersonal coordination across these timescales. We interpret this behavioral shift as a sensorimotor strategy that exploits periodical movements to effectively tune sensory processing in time and allows coping with the disruption in the interpersonal coupling scheme. As such, head movements, which are usually deemed to fulfill communicative functions, may possibly be adapted to help regulate own performance in time.

## Introduction

We adapt to complex and changing environments by finely coordinating multiple body parts at the same time. Doing so “in concert” with others enables very subtle forms of collaboration, such as playing together in a sport team or in a musical ensemble. However, coordinating the self and coordinating with others are most often investigated separately. In effect, motor coordination has long been studied in the sole context of individual actions, while a growing number of studies have recently focused on coordination at the interpersonal level (Sebanz et al., 2006; Schmidt and Richardson, 2008; Laroche et al., 2014; Cornejo et al., 2017; Wiltshire et al., 2020; Dean et al., 2021).

Indeed, similar laws of coordination have been observed in tasks that can be performed by either one or two individuals (e.g., arm movement coordination within or between participants; Amazeen et al., 1995; Schmidt et al., 1998; Schmidt and Richardson, 2008; Fine and Amazeen, 2011). Patterns of coordination emerge even when participants hold opposite intentions or when they are not even aware of their mutual interactions (Issartel et al., 2007; Auvray et al., 2009). In return, patterns at the macro-level constrain the activity of the body parts at the micro-level, inducing synergy among them (Kelso and Engstrom, 2006; Riley et al., 2011).

From this perspective, intrapersonal motor coordination has been hypothesized to be “nested” within higher-order processes of interpersonal coordination (Ramenzoni et al., 2011). A few experiments have testified of the effect of interpersonal processes on intrapersonal coordination. For instance, synchronously walking with another person improved individual gait coordination (Nessler et al., 2015). On the contrary, synchronizing finger tapping with a partner impaired bimanual coordination at the intrapersonal level (Lorås et al., 2019). Most often, interpersonal visual coupling stabilized postural equilibrium (Varlet et al., 2011, 2014; Athreya et al., 2014; Gueugnon et al., 2016). In fact, postural equilibrium can be affected by both intra and interpersonal constraints, with a modulatory effect of task difficulty (Stoffregen et al., 2013).

Earlier studies have found interpersonal coordination to be stronger than intrapersonal coordination when studying them with similar tasks but in separate trials (Schmidt et al., 1998; Black et al., 2007). However, Romero et al. (2015) studied both kinds of coordination simultaneously using a task where one participant had to bring a pointer at the center of a target held by her partner, and observed that interpersonal coupling was stronger than intrapersonal coupling. All in all, these results highlight that intrapersonal coordination can be flexibly subordinated to interpersonal task goals (Bosga et al., 2010). For instance, Aikido experts (but not novices) whom natural movements were artificially perturbed decreased their intrapersonal coordination between sternum, wrist, and elbow in order to strengthen their interpersonal coupling (Caron et al., 2017). To sum up, interpersonal roles and task constraints can thus elicit distinct and complementary modes of intrapersonal coordination (Ramenzoni et al., 2012).

However, previous studies only involved postural or bodily symmetric tasks (ankles during gait, fingers during tapping, arms during precision tasks). Most importantly, they all tackled this issue in dyadic contexts – most often visuomotor tasks – where goals predominantly targeted one level of coordination (either intra- or interpersonal) at the expense of the other. Yet, collaborative activities often involve different body parts (on top of postural demands) that move at distinct paces (unlike synchronized tapping or walking), and they can take place in larger groups of multi-modally coupled individuals whose performances are critical at both intra- and interpersonal levels. More complex and ecological experimental settings are thus required to understand whether and how intrapersonal coordination dynamically adjusts based on changes in interpersonal coupling demands.

A conducted musical ensemble is probably one of the best scenarios to tackle this issue (D’Ausilio et al., 2015; Volpe et al., 2016). Performers coordinate several body parts that move at multiple timescales to play complex musical patterns, and they aim at an exquisite temporal accuracy at both the individual and collective levels of coordination. Coupling between musicians is multimodal (auditory, but also visual, especially with a conductor), and body parts can serve different purposes, from instrumental gestures (those contributing to sound production) to so-called ancillary gestures (such as head movements, which communicate structure and convey expressivity; Nusseck and Wanderley, 2009; Demos et al., 2014).

So far, studies on orchestral ensembles have focused on interpersonal processes (Volpe et al., 2016; Palmer and Zamm, 2017; Wöllner and Keller, 2017; Fadiga et al., 2021). They looked at the coordination between musicians and conductors (D’Ausilio et al., 2012; Meals, 2020), or among musicians, as a function of task difficulty (Badino et al., 2014), leadership (Timmers et al., 2014; Wing et al., 2014; Varni et al., 2019), interpersonal network properties (Shahal et al., 2020), visual communication (Bishop et al., 2021), emotional expression (Chang et al., 2019), or instructions of interactions (Proksch et al., 2022). Solo and collective performances have been compared to quantify behavioral interdependence between players of an ensemble (Papiotis et al., 2014). Such comparisons also helped revealing differences in head motion patterns and functionality across these contexts (Glowinski et al., 2013). In effect, when a player was asked to produce unexpected tempo changes, head movements became more coordinated and asymmetries related to leadership decreased (Badino et al., 2014). The structure of leadership also got weakened together with a decrease in body sway coordination when visual coupling between musicians was removed (Chang et al., 2017). Finally, simply changing the network of visual coupling among players differently affected sensorimotor communication channeled through ancillary (head) and instrumental (bow) movements (Hilt et al., 2019).

Despite providing a relevant context, none of these studies has examined how the interpersonal coupling scheme within a musical ensemble affects the intrapersonal coordination of multiple body parts that music performance involves. To study this, we perturbed the network of sensorimotor communication of an orchestra: the visual coupling of first-section violinists with the conductor (normal condition) was replaced by visual coupling with the second section of violinists (perturbed condition). Because the conductor plays a crucial role in regulating the timing of the players (Luck and Sloboda, 2008; D’Ausilio et al., 2012), preventing vision of his gestures makes it harder for musicians to correctly adjust the timing of their instrumental performance. Given the lack of collectively shared temporal cues provided by the conductor, we expected that violin players would enhance their intrapersonal coordination between ancillary (head) and instrumental (bow) movements to better focus on and regulate the timing of their own instrumental performance.

## Materials and methods

### Participants

A 17-piece orchestral ensemble, with two sections of violinists composed by four players each, and two different conductors were recruited for the experiment. The study was approved by the SIEMPRE Project Management Committee in respect with the standards of the Declaration of Helsinki, and participants gave written informed consent prior to the experimentation. The data set was recorded in the context of the SIEMPRE EU-FP7-FET^1^ project and partially used in a previous publication (Hilt et al., 2019). Here we performed a different pre-processing of the raw data, we computed a different collection of motion features, and we performed different analyses to tackle new research questions.

### Procedure

The members of the orchestra were invited to perform at Casa Paganini in Genova, Italy. This is a research center endowed with a 250-seats auditorium that can be configured to serve as an ecological environment resembling a concert hall. The stage at Casa Paganini is fully equipped with a motion capture system and with professional devices for audiovisual recordings. The orchestra played a familiar excerpt from its repertoire - the opening of “Signor Bruschino” (1813) by Gioacchino Rossini – which eschewed learning effects during the experiment. Furthermore, several features make the piece interesting to study interpersonal coupling processes, as well as their effects on intrapersonal coordination. First, the elevated tempo (near 230 bpm; see Section “Results”) and the speed of execution of passages requiring the bow to revert direction every eighth note place high demands on the fine rhythmical coordination of the players. Second, the important rhythmical differences between the scores of V1 and V2 suits the purpose of evaluating the impact of the presence or absence of a visual coupling between the two sections. Third, the recurrent pauses between the different running passages impose that players pay close attention to their peers and to the conductor in order to finely control their timing when their instrument re-enters the piece. This suits well the goal of studying how changes in visual cues impact the coordination of players’ movement.

To allow the repetition of several takes while avoiding accumulating fatigue over these multiple trials, the chosen excerpt was about 1 min long (i.e., the 55 first bars of the piece). This constitutes a good trade-off between the ecological context of the orchestra and an empirical format where different conditions are examined over repeated measures.

**FIGURE 1:**
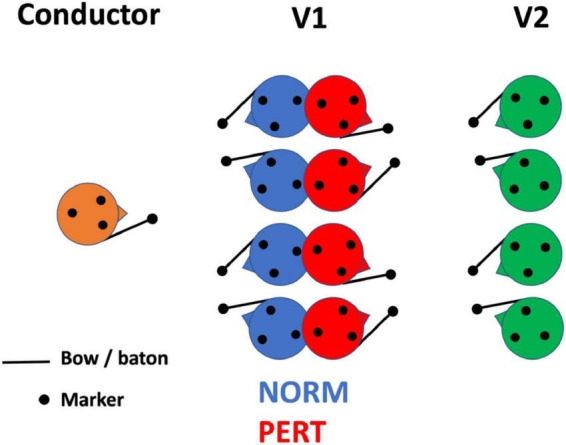
Position of the violinists whose motion has been recorded in normal (NORM) and perturbed (PERT) conditions. The experimental manipulation consisted in changing the arrangement of V1 with respect to the conductor and V2, preventing V1 from seeing the conductor and instead making them face V2.

Importantly, none of the two conductors had practiced with this orchestra before, and they were not given any particular indication regarding their interpretation of the score (e.g., in terms of tempo). Participants completed two experimental conditions: a control condition (NORM) where all performers set at their normal position in the orchestra, and a perturbed condition (PERT) where first-section violinists (V1) turned their back on the conductor and faced the second-section violinists (V2) instead (see Figure 1). This manipulation allowed us to probe the sensitivity of V1’s intrapersonal movement coordination, on which we focus here, to the constraints of their interpersonal coupling^2^. Three takes were recorded with each of the 2 conductors and for each of the 2 conditions (NORM/PERT), leading to a total of 12 takes. To avoid changing the spatial configuration of the orchestra too often and to let the players concentrate on the takes, the experiment was blocked by conductor. More precisely, the first conductor led the orchestra during 3 takes in NORM and then 3 takes in PERT, before the second conductor did the same.

### Apparatus and set-up

Movement data were collected with a Qualisys motion capture system equipped with seven cameras. Violinists and conductors each wore a cap with three passive markers of the Qualisys motion capture system (positioned at Pz, F3, and F4 in the 10–20 electroencephalographic system), and another marker was placed on the tip of their bow and the conductors’ baton. The stability of the cap and the bow was ensured prior to the experimentation. Data tracking was done by the Qualisys Track Manager software, with a sampling rate of 100 Hz. An audio recording of the ensemble was also collected to provide the motor performances with a musical timeline of reference. Synchronized recording and storing of audio and motion capture data was performed by the EyesWeb XMI platform.

### Musical and audio analysis

We analyzed the content of the musical score of V1 as well as V2 in two respects: the articulation techniques being used and the presence of a musical content to be played (as opposed to pauses). The analysis of techniques allowed us to exclude portions of data where performers did not use the bow or used it in an unusual fashion (e.g., a kind of “col legno” where they hit the stand of the desk with the bow, leading to important data losses as the markers got masked in the process). Using video recordings, we also excluded portions where some violinists had to turn the pages of the sheet music. Analyzing the musical content allowed us to exclude from the analysis the portions of data where V1 was not playing. To do so, we segmented the score in steps equivalent to a half-note (or two beats, representing a duration of about 500 ms in this up-tempo piece), and we excluded the steps during which players paused all along. This procedure allowed us to extract four passages of the score in which data could be properly analyzed, and whose duration ranged from 3.5 to 17 s approximately (from bar 1 to the first half of bar 5, from the second half of bar 7 to the first half of bar 11, from the second half of bar 13 to the first half of bar 27, and from bar 39 to bar 55). To extract these passages from the overall time series, we used the audio recordings of the ensemble as referents. Guided by the segmented score, we identified in each trial the starting location of each step by looking for corresponding attack events in the audio signal waveform. To identify the attack portion of a note, we used the software Ableton Live 10, which enables visualization of audio signals at a temporal resolution that is well below the millisecond scale (this task has been performed by the first author who is formally trained and highly experienced in audio micro-editing). Since performances are naturally fluctuating in tempo, this manual annotation also allowed us to estimate tempo locally (for each half-note steps). This will help us relate analysis conducted in the frequency domain to the ongoing tempo of each performance.

### Data pre-processing

Data analyses were performed with custom-made MATLAB codes. We extracted the velocity time series of the head and the bow by computing the Euclidean distance between the successive positions of their associated markers and taking its derivative. The data of the three markers on the cap were averaged to simplify analysis and avoid redundancy. Windows of missing data shorter than 50 ms were cubically interpolated. Longer portions of missing data were considered as absent values (<1% of the data, for the bow motion only). Data of each of the four selected passages were normalized to z-scores and filtered with a zero-phase second-order Butterworth bandpass filter between 0.5 and 12 Hz. The filter bandpass frequencies were chosen based on the main rhythmical values played with the bow and on the minimum length of the passages which prevented from capturing lower frequency components.

## Analysis

### Musical timing information

To better understand the musical timing of the performances, we used the data of the manual segmentation of the audio waveform of the performances. First, we computed the length of each take as the time interval between the beginning of the first half-note segment and the end of the last one. Then, we computed the average tempo of each take. To do this, we computed the average duration of the half-note segments of each take. We divided the results by 2 to obtain the average inter-beat interval duration of each take. Finally, we assessed tempo variability within each take by computing the coefficient of variation (i.e., the ratio of the standard deviation to the mean) of the series of inter-beat interval duration.

### Windowed cross-correlation

To gauge V1’s overall intrapersonal coordination, we looked at the extent to which head and bow motion varied together. To do so, we performed windowed cross-correlations between their respective velocity time series (Boker et al., 2002). We used 1,000 ms windows (approximately four beats, which form a bar in this piece), with a 50% overlap between contiguous windows. Cross-correlation coefficients were computed at up to (±) 60-ms lags. Since the musical score contains eighth notes whose performed rate could be as fast as 120 ms lags larger than half this length would introduce the risk of correlating head and bow motions spanning different notes instead of the same note. Windows were slidden within each of the four selected musical passages separately, and the resulting functions obtained were collapsed for each take of each V1 violinist.

The cross-correlation functions obtained in each 1-s window allowed us to compute 4 statistical indices about the amount of correlation and the lags at which correlation was maximally observed. First, since head and bow motion could have been coordinated with a (variable) delay, we considered the peak correlation across all lags (from −60 to +60 ms in steps of 10 ms). This quantifies coordination strength irrespective of delay. Importantly, Fisher z-transformation was applied to all coefficients before performing any averaging and further statistical analysis. To numerically and graphically present the results, we used hyperbolic tangent transformation to revert the values back to the scale of correlation coefficients. Second, we computed the lags at which peak correlation was observed. This indicates the specific time relationship between head and bow (i.e., whether the head lags ahead or behind the bow). Next, we considered the absolute lag at which peak correlation was observed (independently from its sign). Finally, we estimated lag variability by computing the standard deviation across 1-s windows of the lags corresponding to peak correlation. This was taken as an index of the stability in the coordination pattern between head and bow. These indices were averaged for each take of each V1 violinist before they were submitted to statistical analysis.

### Movement amplitude and spatial dispersion

To better understand the factors that underlie a potential change in the intrapersonal coordination of head and bow, we gauged the amplitude of their displacements. To do so, we first measured the spatial dispersion and the volume these displacements covered in each 1-s windows of analysis and averaged these indices for each take of each V1 violinist (these windows were slidden within each of the 4 selected musical passages separately, before collapsing the results across all windows for each take of each V1 violinist). Spatial dispersion was estimated by computing the mean (Euclidean) distance between all positional datapoints (i.e., all the distances between any two positions in space that head and bow, respectively, visited during each 1-s window). We then computed the volume contained by the 3D convex hull of head and bow spatial trajectories (i.e., the volume of the smallest possible polyhedron that contained all data positions). This indicates the amount of space covered by head and bow motion trajectories.

### Power spectral density

To investigate the temporal structure of bow and head motions, we examined them separately in the frequency domain by computing their respective power spectral density (PSD, using the pwelch function in Matlab). Since the rhythmical content of the musical score is changing over time, and since tempo fluctuates over the course of the performance (with a notable shift toward acceleration in the last portion), we proceeded by short windows. This helped us focusing on the frequency range within which instrumental motion was prominent (between 1 and 8 Hz approximately, which roughly correspond to whole and eighth notes, respectively). We used windows of 3 s (i.e., 300 data points, corresponding to 3 cycles at 1 Hz – the lowest estimated frequency component) within each selected musical passage and with no temporal overlap. To avoid discarding data at the edge of the selected passages due to the windowing procedure, all residual data points not amounting to a 3-s long segment were included in the preceding data window. All windows were then zero-padded to 512 points before estimating PSD.

To verify that spectral content of head and bow motion was meaningfully related to the musical performance, we related the main spectral peaks to the frequencies at which various rhythmical values or metrical levels were performed (whole, half, quarter, and eighth notes). To this end, we took the average tempo at which the piece was performed and computed the relevant harmonics and subharmonics. This helped us to relate peaks observed in the power spectrum with the metrical levels embedded in the performed score (see Section “Results”). Since tempo naturally fluctuates within and between performances, different windows of analysis and trials may yield (slightly) different frequency peaks. Instead of comparing power at fixed frequencies across conditions, we therefore selected the frequencies that corresponded to the similar metrical levels (e.g., the frequencies that corresponded to quarter notes, even though the exact frequencies might slightly differ across trials and windows of analysis). To identify the frequencies that matched metrical levels in each window of analysis and each trial, we relied on manual segmentation of the audio files (see above). Specifically, we averaged the length of the inter-beat intervals that were contained in the corresponding window of analysis, and converted it in a frequency value (i.e., by taking the invert of the interval length expressed in seconds). This gave us an estimate of the frequency associated to the ongoing tempo of the performance. From there, estimates of the frequency associated to other metrical levels could be easily derived (e.g., dividing the frequency value by 4 to obtain the frequency associated to the level of whole notes). Power was then extracted at the frequency bins that locally matched the metrical levels of the performance.

### Power correlation

To verify whether changes in head motion frequency composition could reflect the mirroring of the rhythmical (instrumental) movements of the bow, we constructed time series of the power estimated at each metrical timescale for both head and bow motion. To do so, we took the power estimated at the relevant frequency for each window of PSD analysis (exact frequencies could change across windows; see above). Then, we computed the correlation coefficient between the series of power values obtained across the windows of all four selected musical passages for the head and the bow. Such correlation indicates the extent to which the head and bow motion covaried at each metrical timescale.

To evaluate whether changes in head motion frequency composition could be due to V1 being visually coupled with V2 during PERT, we performed a similar power correlation analysis between V1 head and V2 head or bow motion. To this end, we first computed PSD for V2’s head and bow motion. Then, we repeated the above-described procedure to construct series of power values of V2’s head and bow motion at each metrical timescale. Finally, for each relevant timescale, we computed the power correlation between the head of each V1 performer and the head and bow of the V2 performer each V1 performer was facing. This indicates the extent to which V1’s head motion and V2’s head and bow motion evolved similarly, quantifying the degree of informational coupling between the two sections of violinists.

### Consistency in relative phase

We also evaluated the phase coupling between head and bow across the multiple timescales at which their individual motion was organized. To do so, we apply band-pass filtering (two-pass Butterworth, second order) on 512-points zero-padded data windows (same as used to estimate PSD; see above) with frequency bands defined as ±0.5 Hz relative to each of the musically relevant frequencies (same as defined above). We then applied the Hilbert transform and derived two time series describing the instantaneous phase angle of the head and bow at each relevant timescale. We then took the difference in phase angle between the head and bow (relative phase, RP). By averaging the RP across windows and trials (for each performer separately) we estimated: (1) the vector length (VL) which gauges the stability or consistency of the head-bow phase relationship, (2) the mean angle (MA), which quantifies their mean phase difference, and (3) the mean absolute angle (MAA) which captures the mean phase difference regardless of its directionality (i.e., which series precedes which).

### Statistical analysis

Criteria such as the normality of the distribution and the homoscedasticity of the data could generally not be assumed. Therefore, we used non-parametric Friedman 2-way analyses of variance tests for statistically evaluating differences between conditions (NORM vs. PERT). Given the relatively small sample size (four violinists) which is inherent both to the ecological context of the experiment and to the actual composition of a (chamber) orchestra, Friedman test offers the opportunity to directly address the difference between our two conditions of interest (NORM and PERT) at the level of the section while controlling for the effects linked to the subjects’ factor. For all analyses, the data obtained for the two different conductors were collapsed. We checked that the conductor type did not introduce major differences in the main findings (i.e., the overall intrapersonal coordination measured with windowed cross-correlations). Since we observed some differences for other analyses, we also report the comparisons between conductors for all variables and for each experimental condition in the Supplementary material. For the main analyses, collapsing data across conductors resulted in matrices of four blocks (corresponding to four violinists) that each contained six repeated measures, and two experimental conditions (NORM vs. PERT) as the column effect to be tested.

## Results

### Similar musical timing across conditions

The average length of the excerpt was 57.8 ± 1.5 s and was similar across conditions (NORM: 57.8 ± 1.8 s; PERT: 57.8 ± 1.2 s). The average inter-beat interval duration was 263 ± 7 ms, which is equivalent to 229 bpm, and was similar across conditions (NORM: 263 ± 8 ms; PERT: 263 ± 6 ms). The coefficient of variation of inter-beat interval duration was 0.062 (±0.011). It was slightly higher in NORM (0.064 ± 0.015) than in PERT (0.0596 ± 0.005). Using a Friedman test with the two conductors as subject factor with three takes each, this difference was not significant (chi2 = 0.38; *p* = 0.53). Overall, musical timing information such as average tempo and tempo variability did not differ across conditions.

### Head and bow show enhanced (intrapersonal) coordination during perturbation

**FIGURE 2:**
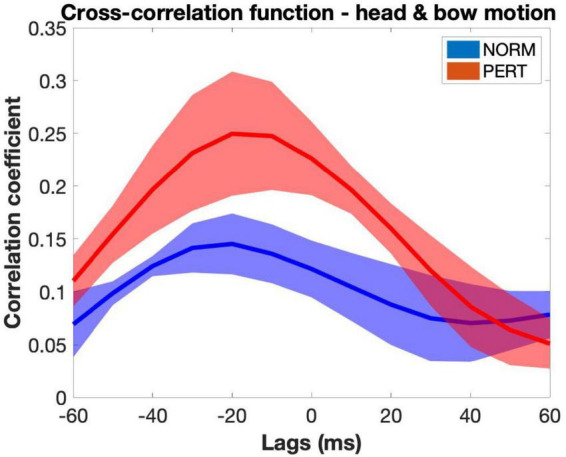
Average cross-correlation functions between head and bow motion across takes and violinists in the NORM and PERT conditions (shaded areas represent standard errors of the mean). Correlation coefficients were higher in the PERT condition than in the NORM condition, suggesting that head and bow were more strongly coupled during PERT. In both conditions, average functions peak at negative lags, indicating that head movements slightly preceded bow movements. Note that this figure represents all cross-correlation functions computed for each 1-s window averaged across participants and then across takes. The amplitude of the peaks of these average functions and the lag at which they are observed can thus differ from the values of the indices obtained by extracting only the peak correlation value and the associated lag in each 1-s window.

**FIGURE 3:**
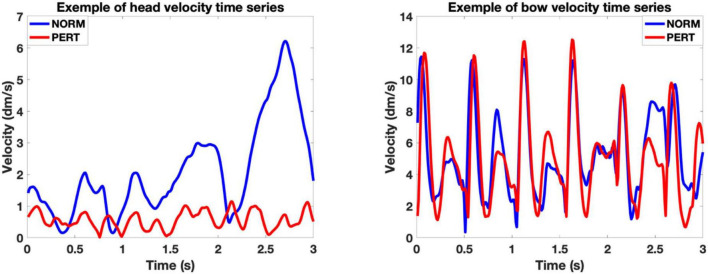
Representative examples of head **(Left)** and bow **(Right)** velocity time series. The examples correspond to a 3-s window of the movement of the first violinist of V1, during the third take in NORM and the third take in PERT with the first conductor, starting at bar 18. In these examples, we can appreciate the periodical nature of both head and bow motion, the similarity of bow motion across conditions and the differences in amplitude and frequency composition of head motion across those conditions. Notice that the Y-scales of the examples of head and bow motion differ, as the possible range of head motion is physiologically restricted compared to the amplitude required by the execution of bow motion.

To gauge V1’s overall intrapersonal coordination, we performed windowed cross-correlations on head and bow velocity time series (average cross-correlation functions are presented in Figure 2, and representative examples of velocity time series are presented in Figure 3). Peak coefficients were significantly higher in PERT (*r* = 0.42, ±0.07) than in NORM (*r* = 0.36, ±0.05; chi2 = 12.41; *p* = 0.0004). Head and bow thus tended to move more similarly during PERT, irrespectively of the lag difference between the two time series. On average, peak correlations were observed near, yet slightly before lag-0 (NORM: −3.19 ± 9.57 ms; PERT: −7.08 ± 8.29 ms), indicating that performers tended to synchronize their head and bow movements, yet head movements slightly preceded bow movements. Lags associated to peak correlations did not significantly differ across conditions (chi2 = 0.41, *p* = 0.52). However, there was a marginal tendency for absolute lags to be smaller in PERT (32.92 ± 9.90 ms) than in NORM (38.89 ± 8.33 ms; chi = 3.41; *p* = 0.0647). Thus, head and bow tended to move more synchronously during PERT than NORM. Finally, the lag at which peak correlations occurred was less variable in PERT (33.63 ± 11.62 ms) than in NORM (42.09 ± 8.65 ms; chi2 = 5.77; *p* = 0.0163). The temporal coordination between head and bow motion was thus more stable (i.e., less variable) during PERT. In sum, head and bow overall intrapersonal coordination was stronger (higher peak correlation coefficients), more in phase (closer to lag-0) and more stable (less variability in the lags of peak correlations) during PERT. However, these results only provide us with hints about the overall similarity of variations between head and bow velocity at the 1-s window scale. Finer-grained analyses are required to parse the effects of the different timescales at which periodical variations were observed (see Figure 3). This issue is treated further below, with the study of the frequency composition of the movement and the analyses of phase relationships at the different frequency components it highlighted.

### Head (but not bow) motion is reduced in amplitude during perturbation

To investigate the kinematic changes that underlie differences in intrapersonal coordination, we gauged movements amplitude by measuring their spatial dispersion (mean inter-distance between all datapoint positions) and the volume covered by head and bow motion (convex hull).

**FIGURE 4:**
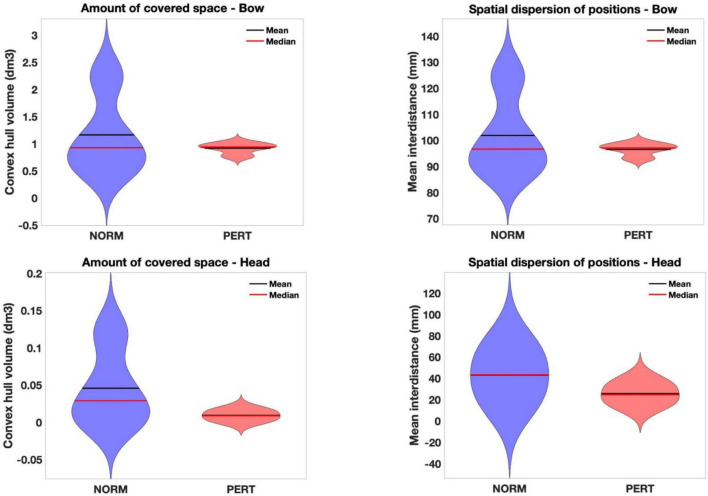
Violin plots of the amount of covered space (convex hull volume – **Left**) and spatial dispersion (mean inter-distance – **Right**) of bow **(Upper)** and head **(Lower)** positions in each experimental condition. Head (but not bow) motion was drastically reduced in amplitude during perturbation. This partly explains the reduced variability in that condition, although it might also reflect more stable and shared movement strategies. In effect, while these variables didn’t differ in magnitude across conditions for the bow motion, variability among players was also drastically reduced during perturbation. Notice that the Y-scales for head and bow motion differ, because the possible range of head motion is physiologically restricted compared to the amplitude required by the execution of bow motion.

Mean inter-distances did not differ across conditions for the bow [chi-2 = 2.91(*10e-30); *p* = 1] but they were significantly larger in NORM than in PERT for the head (chi-2 = 23.08; *p* = 0.000002; see Table 1 and Figure 4). Differences in variance across conditions are important, but individual comparisons confirmed that the reduction of spatial dispersion during PERT was observed for all players (with differences ranging from a decrease of 8% for the player that moved with the least amount of spatial dispersion to a decrease of more than 50%). Head (but not bow) motion trajectory thus visited positions that were less spread in space during PERT.

**TABLE 1:** Mean inter-distance (in mm) between positional datapoint and convex hull volume (in cm^3^) indicating head and bow trajectories, compared across experimental conditions.

	Mean inter-distance		Convex hull	
	Bow	Head	Bow	Head
NORM mean (std.)	101.86 (16.11)	43.00 (24.15)	1162.69 (764.14)	45.727 (53.95)
PERT mean (std.)	96.52 (2.52)	25.62 (10.15)	916.47 (106.23)	9.308 (7.27)
Chi-2	2.9127e-30	23.08	0.06	25.44
*P*-value	1.00	**0.000002**	0.8102	**0.0000005**

*P*-values in bold indicate significant differences.

The volume contained in the convex hull of bow trajectories did not differ between conditions (chi-2 = 0.06; *p* = 0.81), but it was significantly smaller in PERT than in NORM for head trajectories (chi-2 = 25.44; *p* = 0.0000005; see Table 1 and Figure 4). Motion of the head, but not of the bow, covered smaller portions of space during PERT. Interestingly, mean inter-distances and convex hull of both the head and the bow were more variable across performers in NORM than in PERT (see Figure 4). Individual motor strategies were thus sparser in NORM and more commonly shared in PERT. However, smaller convex hulls during PERT were observed for all players, with decreases ranging from 28 to 82%. In sum, movement amplitude and dispersion of the head were smaller in PERT than in NORM, but they did not vary across conditions for the bow (the representative examples of head and bow velocity time series in Figure 3 illustrate these differences – or lack thereof – well).

### Frequency composition of head motion matches the score metrical hierarchy during perturbation

**FIGURE 5:**
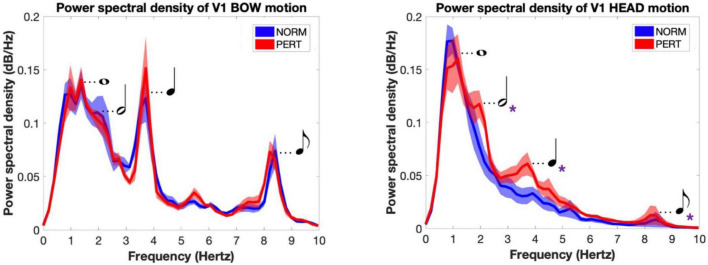
Power spectral density of V1’s bow **(Left)** and head **(Right)** motions for each experimental condition. Main frequency peaks are associated with the rhythmical values they corresponded to in the performed piece (and represented here as musical notations). Bow motion was characterized by multiple periodicities that reflected the metrical organization of the score. Head motion shift from simple patterns dominated by one frequency peak near 1 Hz in NORM to more complex patterns of motion in PERT, where multiple, faster periodicities appeared and matched those found in the bow motion. Shaded areas represent standard errors of the mean; violet asterisks denote the metrical scales where power was significantly different across conditions.

The average spectrum of the bow velocity showed multiple peaks around 1, 2, 4, and 8 Hz (peaks observed at these frequency components will henceforth be designated as P1, P2, P3, and P4, respectively; see Figure 5). According to the segmentation we performed on audio tracks, whole, half, quarter, and eighth notes were performed at rates of 0.96, 1.93, 3.85, and 7.70 Hz, respectively. The overall spectral composition of bow motion thus reflected the rhythmical organization of the performance well, with multiple frequency components corresponding to the different metrical timescales of the score.

To compare frequency peaks across conditions, we extracted power at the frequency bins corresponding to each of the four metrical timescales described above. No difference was found across conditions for any of the peaks in the bow motion (Table 2). In other words, the bow movements closely mirrored the metrical organization of the score rather than being affected by the nature of interpersonal coupling.

**TABLE 2:** Power values (db/Hz) extracted from the power spectral density spectra at the four frequency bins that corresponded to musical metrical levels (P1 – P4), compared across experimental conditions for the motion of the bow.

BOW	P1	P2	P3	P4
NORM mean (std.)	0.125 (0.031)	0.109 (0.027)	0.152 (0.065)	0.079 (0.026)
PERT mean (std.)	0.133 (0.043)	0.108 (0.013)	0.147 (0.067)	0.094 (0.022)
Chi-2	1.64	0.16	0.03	2.83
*P*-value	0.2002	0.6889	0.8728	0.0927

In contrast to bow movements, head movements clearly differ between NORM and PERT. In NORM, the frequency composition of head movements was much simpler than for bow movements (Figure 5). The spectrum is dominated by a main component centered around 1 Hz (P1) – i.e., the frequency that corresponded to the average periodicity of the bar, or whole note, during the performances. During PERT, this peak persisted (although peak frequency shifted slightly higher to 1.2 Hz), but other peaks appeared around 2.0 Hz (P2), 3.7 Hz (P3), and 8.2 Hz (P4). Just like those of the bow motion, these additional peaks matched the average frequency of the metrical timescales of the performed piece well (half, quarter, and eighth notes). Power observed at these additional peaks (i.e., P2, P3, P4, but not P1) was significantly higher during PERT compared to NORM (Table 3). During PERT, head motion thus became more complex, displaying activity at multiple and faster metrical timescales than during NORM (see Figure 3 for a representative example of changes in the rhythmical patterns of head motion). Further, in PERT more than in NORM, the overall frequency composition of head motion resembled that of the bow motion.

**TABLE 3:** Power values (in db/Hz) extracted from the power spectral density spectra at the four frequency bins that corresponded to musical metrical levels (P1 – P4), compared across experimental conditions for the motion of the head.

HEAD	P1	P2	P3	P4
NORM mean (std.)	0.173 (0.024)	0.096 (0.047)	0.033 (0.017)	0.007 (0.010)
PERT mean (std.)	0.153 (0.051)	0.117 (0.033)	0.060 (0.014)	0.013 (0.018)
Chi-2	2.83	5.77	14.16	12.98
*P*-value	0.0927	**0.0163**	**0.0002**	**0.0003**

Bolded *P*-values indicate significance (*p* < 0.05).

### Spectral power does not show local intra- (head-bow) and inter- (V1–V2) personal correlation

To check whether the frequency composition of head motion during PERT reflected the mirroring of the bow rhythmical movements, we computed the correlation between power observed in head and bow of V1 at each relevant metrical timescale. Correlation coefficients were very small, and no significant difference was found between conditions for any of the timescales (Table 4). Whereas multiscale head motion patterns reflected the overall metrical structure of the score more during PERT, they did not match local rhythmic variations of the bow.

**TABLE 4:** Correlation coefficients representing the co-evolution of V1’s head and bow power spectral density at the four frequency bins that corresponded to the piece metrical levels (P1 – P4), compared across experimental conditions.

	P1	P2	P3	P4
NORM mean (std.)	−0.01 (0.13)	−0.1 (0.24)	0.06 (0.16)	0.44 (0.25)
PERT mean (std.)	−0.03 (0.08)	0.03 (0.28)	0.19 (0.28)	0.55 (0.33)
Chi-2	0.1	4.01	1.26	2.08
*P*-value	0.7488	**0.0453**	0.2623	0.1495

Bolded *P*-values indicate significance (*p* < 0.05).

**FIGURE 6:**
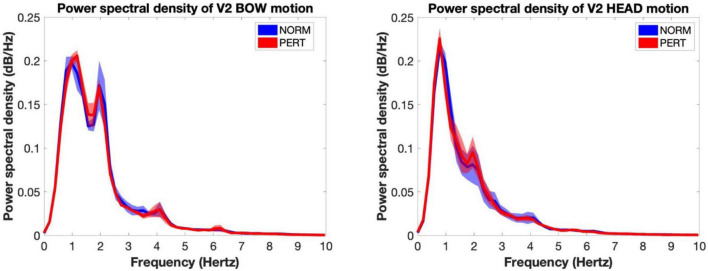
Power spectral density of the motion of the bow **(Left)** and the head **(Right)** of V2 for each experimental condition (shaded areas represent inter-subject standard error). The frequency composition of the bow motion was concentrated around 1 and 2 Hz (reflecting whole and half notes) with peaks near 4 and 8 Hz (quarter and eighth notes). The motion of the head as dominated by a component situated near 1 Hz, with an important secondary component near 2 Hz and a tiny peak near 4 Hz. No change was observed across conditions. Importantly, this frequency composition hardly explains the shift observed in V1 during perturbation, when they were facing V2.

To verify whether the changes in head motion frequency composition observed during PERT were due to the visual coupling with V2, we first computed the PSD of V2’s head and bow motion, and then we computed the correlation between power observed in V1’s head motion and V2’s head as well as bow motion at each relevant metrical timescale. In both conditions, the average spectrum of V2’s bow motion mainly featured peaks close to 1 and 2 Hz (whole and half-notes, or P1 and P2), with additional small peaks around 4 Hz (quarter-note level, P3) and 6 Hz (dotted eighth notes; see Figure 6). V2’s head motion was dominated by a frequency component close to 1 Hz (P1), with a smaller peak around 2 Hz (P2) and a small hump around 4 Hz (P3). Besides P2, which was present in V2’s motion and enhanced during PERT in V1’s head motion, the frequency composition of V2’s head and bow motion hardly reflected the overall changes observed in V1’s head motion during PERT, where the two sections faced each other. Correlation coefficients between V1 and V2 power time series were also very small, and no difference was observed between conditions for any of the peaks (Table 5). Therefore, the evolution of the frequency composition of V1’s head motion does not seem to be informed by V2’s head or bow motion.

**TABLE 5:** Correlation coefficients representing the co-evolution of V1’s and V2’s head (upper table) and V1’s head and V2’s bow (lower table) power spectral density at the four frequency bins that corresponded to the piece metrical levels (P1 – P4), compared across experimental conditions.

	P1	P2	P3	P4
**HEAD**				
NORM mean (std.)	0.05 (0.23)	0.08 (0.09)	0.22 (0.21)	−0.03 (0.06)
PERT mean (std.)	−0.08 (0.15)	−0.09 (0.03)	−0.03 (0.11)	0.06 (0.11)
Chi-2	1.44	1.08	1.44	0.16
*P*-value	0.2298	0.2980	0.2298	0.6889
**BOW**				
NORM mean (std.)	0.03 (0.23)	−0.07 (0.17)	0.18 (0.38)	0.10 (0.14)
PERT mean (std.)	0.08 (0.14)	−0.10 (0.07)	0.02 (0.18)	0.30 (0.37)
Chi-2	1.26	0.03	0.1	2.56
*P*-value	0.2623	0.8728	0.7488	0.1093

### Intrapersonal coordination increased differentially at multiple timescales

**FIGURE 7:**
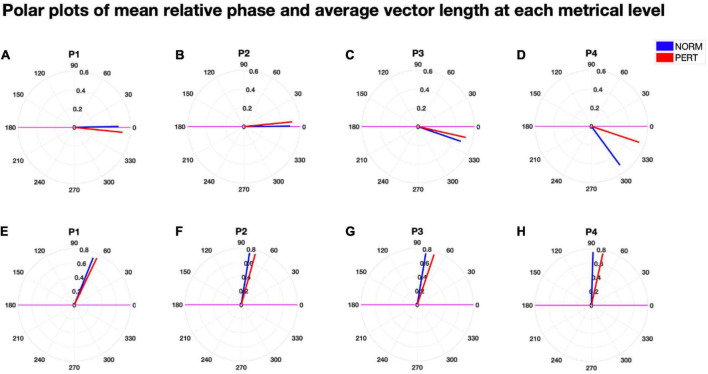
Polar plots of the (signed) mean relative phase **(A–D)** and the mean absolute relative phase **(E–H)** as well as their associated vector length, for each experimental condition and at each metrical timescale. The size of the vector length of the (signed) mean relative phase was higher in PERT than in NORM, and significantly so for P1 and P3. The (signed) mean relative phase was significantly shorter (i.e., closer to 0°, that is, synchrony) in PERT than in NORM for P4. The mean absolute relative phase was also shorter in PERT than in NORM for P2, P3, and P4. Overall, phase relationships were thus slightly but significantly more stable and closer to synchrony at several metrical timescales in PERT than in NORM.

To evaluate head and bow coupling with respect to the multiscale nature of their movements, we analyzed their phase relationships at each metrical timescale (see Figure 7). Consistency in the phase relationship was higher in PERT than in NORM at all timescales, and significantly so at the level of the bar (P1: Chi2 = 8.31, *p* = 0.0039) and the beat (P3: Chi2 = 5.3, *p* = 0.025; see Table 6). However, the difference at P3 was mainly driven by the performance with conductor 2 (see Supplementary material). There was also a marginal trend for vector length to be higher in PERT than in NORM at P2 for conductor 1 only (see Supplementary material). All in all, the coordination between head and bow motion was therefore more stable in PERT but at selective metrical levels that dominated bow motion frequency composition the most. Mean phase differences were close to zero degree (i.e., in-phase) for P1 and P2 and slightly negative for P3 and P4 (i.e., head motion shortly preceded the bow motion). Mean phase differences were comparable between NORM and PERT except that for P4 (Chi2 = 4.33, *p* = 0.0374; see Table 6). This indicates that, at the eighth-note level, head and bow motion were more in-phase during PERT. Mean absolute phase differences were smaller in PERT than in NORM, and the difference was significant for P2, P3, and P4 (P2: Chi2 = 4.33, *p* = 0.0374; P3: Chi2 = 9.75, *p* = 0.0018; P4: Chi2 = 10.78, *p* = 0.001; see Table 6, middle). Head and bow were thus moving more in-phase during PERT at all metrical timescales except that at the whole-note level (P1).

**TABLE 6:** Vector length (upper table), mean relative angle (in radians, middle table) and mean absolute angle (in radians, lower table) of the relative phase between head and bow motion, computed at each metrical timescale (P1 – P4), compared across experimental conditions.

	P1	P2	P3	P4
**Vector length**				
NORM mean (std.)	0.473 (0.039)	0.493 (0.057)	0.482 (0.023)	0.514 (0.076)
PERT mean (std.)	0.517 (0.046)	0.518 (0.037)	0.521 (0.014)	0.537 (0.091)
Chi-2	8.31	1.85	5.3	1.85
*P*-value	**0.0039**	0.1735	**0.025**	0.1735
**Mean angle**				
NORM mean (std.)	0.022 (0.258)	0.011 (1.350)	−0.331 (0.560)	−0.937 (1.088)
PERT mean (std.)	−0.102 (0.343)	0.098 (0.931)	−0.223 (0.448)	−0.323 (0.866)
Chi-2	1.85	2.56	0.01	4.33
*P*-value	0.1735	0.1093	0.9362	**0.0374**
**Mean absolute angle**				
NORM mean (std.)	1.190 (0.062)	1.411 (0.141)	1.405 (0.109)	1.539 (0.090)
PERT mean (std.)	1.123 (0.112)	1.300 (0.160)	1.246 (0.106)	1.352 (0.179)
Chi-2	2.08	4.33	9.75	10.78
*P*-value	0.1495	**0.0374**	**0.0018**	**0.001**

Bolded *P*-values indicate significance (*p* < 0.05).

## Discussion

During sensorimotor interactions, people tend to coordinate their movements interpersonally and beyond intention or awareness (Issartel et al., 2007; Auvray et al., 2009). Interpersonal interactions can thus constrain and shape individual behavior (De Jaegher et al., 2010). Yet, little is known about how sensitive the intrapersonal coordination of multiple body parts is to interpersonal coupling constraints, especially in complex ecological settings. To study it, we chose to make a trade-off between the ecological context of an orchestra playing an excerpt of a familiar piece of its repertoire and an empirical format where different conditions were observed across repeated measures. Specifically, we replaced the visual coupling of first-section violinists with the conductor (normal condition) by a visual coupling with the second section of violinists (perturbed condition). Focusing the analysis on the intrapersonal coordination of head and bow movements allowed us to gauge its sensitivity to varying interpersonal coupling constraints.

We observed three main effects in first-section violinists: (1) as expected, the overall intrapersonal coordination of head and bow motion increased, (2) qualitative shifts occurred in head (but not bow) movements: they diminished in amplitude but increased in spectral complexity, to reflect more closely the metrical structure of the score, and (3) the intrapersonal coordination of head and bow movements increased differentially at multiple timescales. We will discuss each of these results in the following.

In both conditions, first-section violinists’ head and bow motion were weakly but non-randomly coupled, illustrating the soft entrainment of ancillary movements to instrumental gestures (Colley et al., 2020). Interpersonal coupling constraints, however, affected head and bow intrapersonal coordination. Perturbation of first-section violinists’ visual coupling network increased the coordination between their own head and bow in terms of strength (larger peak correlation values), temporal tightness (shorter lags at which peak correlations were observed), and stability (increased consistency in the lags at which peak correlations occurred). This resonates with studies on joint-precision tasks showing that intrapersonal coordination increases with task difficulty (Ramenzoni et al., 2011; Davis et al., 2017). In contrast, Aikido experts decreased their intrapersonal coordination to strengthen their interpersonal coupling (Caron et al., 2017). In the latter experiment, however, and differently from the present study, perturbation was applied to individual properties of movement (using arm weights), while the task-goal was interpersonal in nature (coordinating defense and attack moves). What is common to all these observations is the apparent flexibility with which participants can modulate their intrapersonal coordination when they have to cope with changes in interpersonal task constrains.

What is new here is that the effect of perturbation was not merely quantitative (in contrast to differences in variability reported previously, or to the overall increase in intrapersonal coordination in the present study). Rather, a shift toward a different, more complex pattern of coordination occurred during perturbation. In the normal condition, head motion was dominated by a single frequency component at the level of the bar (or whole note). This reflects the tendency of postural sways (which are embedded into head motion) to embody the temporal structure of musical performances (Nusseck and Wanderley, 2009; MacRitchie et al., 2013; Demos et al., 2018; same as during music listening: Burger et al., 2018). During perturbation, however, head movements shifted toward a more complex regime, introducing multiple and faster frequency components. These components were situated at harmonic ratios (near 2, 4, and 8 Hz) of the fundamental frequency observed in normal condition (near 1 Hz). Appearances of harmonic peaks have previously been reported in expert motor learning (Cordier et al., 1996) as well as in power spectra of phase relationships during simple bimanual coordination (Fuchs and Kelso, 1994). These changes were attributed to modifications in the intrinsic dynamics governing movement and might not necessarily reflect a genuine periodical activity at these frequency bands. However, the visual inspection of the time series (see Figure 3 for a representative example) seems to indicate the genuine presence of faster periodical components in head motion. Importantly, these additional components were also observed in the bow motion regardless of the condition, and they well reflected the metrical organization of the musical score (half, quarter and eighth notes). This suggests that these frequency components resulted from changes in the rhythmical patterns with which V1 players moved their head during perturbation: patterns that more closely matched the metrical hierarchy of the piece.

Changes in head motion could reflect a communicative strategy aiming at fostering interpersonal coordination during perturbation (Davidson and Good, 2002; Glowinski et al., 2013). Indeed, visual coupling suffices to induce interpersonal coordination (Richardson et al., 2007). More generally, expressive gestures enhance visuomotor entrainment (Coorevits et al., 2020). A familiar musical example is players nodding their head to cue the beat (Bishop and Goebl, 2018). Thus, musicians synchronize head movements more during unstable moments or when the auditory feedback of their partner is compromised (Goebl and Palmer, 2009; Badino et al., 2014; Bishop et al., 2019; see also Hadley and Ward, 2021 for similar observations in the context of conversations). Importantly, players move more when they seek more interaction with their partners but move their head less when visuomotor communication is reduced or hindered (Bishop et al., 2019, 2021). Here, the amplitude of V1 head motion decreased drastically during perturbation. This is unlikely to indicate an attempt to increase visuomotor communication with V2. However, the reduction in amplitude might be a consequence of the changes in the frequency composition of head motion. Indeed, the addition of periodic motion at higher frequencies requires more direction reversals and acceleration breakpoint, drastically reducing the possible range of movement amplitude.

Changes in head motion could also reflect interpersonal entrainment to the second section of violinists (Hilt et al., 2019). However, the frequency composition of the second section’s movement did not vary across conditions in the same way as it did for the first section, ruling out the possibility that spectral changes in V1 are the effect of entrainment to V2. One could also question the role of the vision of the conductor and its absence during perturbation. The frequency composition of the conductors’ motion barely changed across conditions and poorly matched the pattern of V1 head motion, even in normal condition (see Supplementary material). In effect, for both the conductors’ baton and head motion, the dominant frequency component was situated near 2 Hz. An important peak was also observed at 1 Hz in the conductors’ head motion, as well as a clear peak near 4 Hz in the baton motion, but only in one of the two conductors. Plus, differences in the frequency composition of movement across conductors did not affect the frequency composition of V1 head motion: only the experimental manipulation did. More generally, differences across conductors were small. Each conductor having led the orchestra in two separate, consecutive blocks, this indicates that motor performances and differences across conditions were rather stable across time, and that the coupling with the conductor (or its absence) hardly accounts for the frequency composition of V1 head motion.

Increased intrapersonal coordination and changes in head motion might rather reflect individual strategies to cope with the introduction of challenging interpersonal coupling constraints. In line with this interpretation, similar effects have been observed in solo string players during various forms of perturbations. First, enhanced metrical coupling between head and bow motion, reduced head motion and shifts toward movements at faster metrical timescales all occurred spontaneously and without much change in bow motion properties when cellists had their posture constrained (Rozé et al., 2017, 2019, 2020). Next, violists’ head motion changed (and most often diminished) when their bow strokes were constrained as well (Visi et al., 2014). Furthermore, adapting to a metronome also decreased the upper-body movements of violinists, but increased motion in their sacrum, whose usual stability supports upper-body expressivity (Glowinski et al., 2014). Spontaneous compensation between body parts thus seems to help string players to flexibly shift motor strategies (Shan et al., 2012; Verrel et al., 2014). In short, the reorganization of movements across body parts allowed players to cope with intrapersonal constraints in previous studies, and therefore probably helped to cope with interpersonal ones in the present study.

According to our initial hypothesis, enhanced head and bow coupling could reflect an attempt to stabilize motor coordination. This is coherent with the observation that during perturbation, head motion matched bow motion more closely from a metrical point of view. Nonetheless, this required head motion to increase in complexity. In line with this observation, it has previously been reported that greater intrapersonal coupling can be accompanied by more complexity when a joint task increases in difficulty (Davis et al., 2017). In effect, stabilizing interpersonal coordination sometimes requires the recruitment of additional degrees of freedom (Fine et al., 2013; Fine and Amazeen, 2014). However, the frequency composition of head motion only reflected bow motion at the scale of the whole excerpt. In effect, when examined at a finer-grained scale through power correlations, head and bow motion frequency composition appeared to fluctuate independently across the piece. This suggests that during perturbation, head movements were marking the metrical organization of the score rather than (anticipatively) mirroring the rhythmical performance of the bow. This argues against a purely, and rigid, synergistic motor strategy.

To interpret these results, we probably need to consider the tight link between action and perception and the role that the former plays for the latter (Varela et al., 1991; O’Regan and Noë, 2001). While head movements can express the perception of musical forms (Colley et al., 2020), body movements also actively contribute to perceptual experiences (Noë, 2004; Di Paolo et al., 2017; Benedetto et al., 2020). Especially, motor activity and auditory processes hold strong links (D’Ausilio et al., 2006; Zatorre et al., 2007; Morillon and Baillet, 2017; Froese and González-Grandón, 2020). For instance, movements can entrain to auditory rhythms with clear benefits on rhythm perception (Todd et al., 1999; Su and Pöppel, 2012). In particular, head motion stimulates the vestibular system which knowingly contributes to beat and meter perception (Phillips-Silver and Trainor, 2005, 2008). This phenomenon can be modeled as an oscillatory motor network that is entrained to the musical rhythm, and entrains, in turn, an auditory network, eventually improving the processing of incoming acoustic information (Tichko et al., 2021). Head movements can thus help to appropriately tune the auditory system to the ongoing rhythmical and metrical structures, playing a role in the very perception of musical events (and not merely expressing or reflecting such perceptual process).

The temporal coordination between movements and auditory processes is rather fine-grained: for example, fluctuations in auditory sensitivity are phase-aligned to simple periodical movements (Morillon and Baillet, 2017; Zalta et al., 2020). This resonates with the Dynamic Attending Theory which holds that attention is tuned to the temporal structure of sensory events, thereby enhancing sensory processing at specific points in time when salient/relevant events are expected (Large and Jones, 1999). If overt movement improves the tuning of attentional fluctuations over time, then V1 head movements may aid in framing auditory processing in accordance with the musical structure they mark. Miyata et al. (2017; 2018; 2021) have shown that interpersonal visual coupling with others affect individual audio-motor coordination, but that interpersonal auditory coupling had compensatory effects when vision degraded individual audio-motor performances. In our study, V1 players might thus have exploited the link between head motion and auditory processes in order to focus their perceptual activity on the auditory stream, thereby compensating for the perturbation of their habitual visual coupling with the conductor. This would explain the increased strength and synchronicity of intrapersonal temporal coordination during perturbation. Moving the head more in phase with the bow would help focusing on note onsets and locating them more accurately in time. Faster periodicities of head movement should increase the frequency and saliency of attentional checkpoints, while smaller movements should sharpen the temporal focus of attentional pick-ups. In effect, smaller movements reflect a deeper focus on note playing accuracy, shifting the attention away from the interaction with co-performers (Bishop et al., 2019). This would explain why the shortening of the phase lag between head and bow motion was most visible at the highest frequency component (around 8 Hz, where not only the absolute but also the relative phase lag decreased significantly during perturbation). Interestingly, head and bow motion frequency composition evolved most similarly at that one particular metrical level. Moving the head at the highest frequency thus accompanied musically dense passages, probably enabling a narrower focus of attention that fits rapid changes of notes.

During perturbation, however, head movements did not just increase in frequency: they became more complex as multiple frequency components appeared. Similarly, musical events are not merely periodical but rather organized at multiple timescales, forming a structure that span several metrical levels. The perception of this metrical framework can be modeled as an entrainment of neural oscillations to auditory events at multiple timescales (Large and Snyder, 2009), and provides a prism through which music can be flexibly attended to (Keller, 2001). This flexibility allows to shift the focus of attention between different timescales of organization of auditory events (Nolden and Koch, 2017). Musicians can then exploit this ability by mentally foregrounding those metrical levels that best help to cope with momentary goals or constraints – for instance, concentrating on the quarter-note level when the group lacks coordination (Berger, 1997).

The metrical framework through which we attend to music is reflected in body movements as well: we spontaneously move at timescales that match those of the metrical organization of music we interact with, whether as listeners (Toiviainen et al., 2010), dancers (Leman and Naveda, 2010), or performers (Walton et al., 2015; Eerola et al., 2018). However, these patterns of movement are not the mere expression of music perception, but they rather seem to play an active role in the constitution of perceptual experiences. For instance, shifting movements across metrical levels impacts how time is perceived in return (Hammerschmidt and Wöllner, 2020; Wöllner and Hammerschmidt, 2021). Body movements thus actively tune the metrical framework through which we attend to music (Large et al., 2015; Kozak, 2021). In violinists, the link between body kinematics and focus of attention has been demonstrated as well (Allingham et al., 2021). More particularly, shifts in head motion patterns during perturbation of solo string players have been interpreted as reflecting attentional changes (Visi et al., 2014; Rozé et al., 2020). Interestingly, cellists too moved their head more frequently and with more energy at several timescales (especially at half and quarter-notes levels) when they played a melody by focusing their attention on shorter rather than longer groupings of notes (Huberth and Fujioka, 2018). In fact, even spectators sense that the timescales at which musicians move their body reflect the way they attend to their own performance: they attribute slower body sways to communicative intents, and faster head nods to pulse perception (Eerola et al., 2018).

In our experiment, changes in head motion during perturbation might thus not only reflect an increase in perceptual focus but also a qualitative reframing of its temporal organization. This reorganization consisted of bringing more diverse and higher-frequency metrical levels into focus, possibly to concentrate on and regulate the short-term timing of the performance. This would explain why head and bow motion were more synchronous at multiple timescales during perturbation. This would also explain why the stability of head and bow coordination increased at the functionally most relevant timescales, namely, the metrical levels of the bar and the beat that dominated bow motion (although, in the case of the beat, this was true with only one of the conductors). In short, shifting toward patterns where the head moves at multiple timescales should have allowed players to frame, hierarchize and shift the temporal organization of perceptual focus across metrical levels. This reframing could then have been used as a background perspective against which coordination of incoming sensory events (the performance of the self and others) was accurately monitored, gauged and ultimately regulated, allowing violinists to better cope with the lack of timing cues from the conductor during perturbation.

## Conclusive remarks, limits and future directions

When their network of interpersonal sensorimotor communication was perturbed, first-section violinists increased their intrapersonal coordination and changed their head motion patterns. The present study thus highlights the sensitivity of intrapersonal body coordination to interpersonal coupling constraints in the complex and ecological context of a musical ensemble. By showing how flexible the coordination between body parts is, our results also underscore the (multi-)functional role of non-instrumental gestures such as head movement. This questions the conceptual segregation between ancillary and instrumental movements. In effect, posture and head movements seem to offer support for the control of instrumental gestures (Rozé et al., 2020). By shaping attention, framing sensory processing, and thereby honing musicians’ sense of timing, ancillary movements might directly participate to the fine-grained motor coordination of instrumental gestures (Colley et al., 2020). This echoes the fact that the control of such movements and musical learning co-develop (Rodger et al., 2013). Head movements would thus not only constitute a way to communicate with others, but also a strategy to inform the very self. This highlights the importance of studying intrapersonal and interpersonal coordination processes in the context of each other, and at multiple timescales.

An important limitation of these results is that they have been observed in one peculiar context: a short excerpt of one particular piece, which in the perturbation condition was performed in an unusual configuration. First, the short duration of the excerpt is a clear limitation because interpersonal coordination processes can evolve over time and can take time to fully form. In return, longer periods of interpersonal coupling might change the way intrapersonal coordination is impacted. Nonetheless, this short duration simultaneously provided us with an opportunity to zoom in the moments where interpersonal coordination had yet to be established, and where intrapersonal coordination could particularly support this endeavor and/or to compensate for the perturbation of the habitual visual coupling.

Second, it is possible that the specific musical demands of the piece encouraged the behavioral phenomena we observed. The tight control of the timing required by the speed of execution, the repeated need to re-enter the piece, and the large differentiation with the rhythmical content of the score of V2 might have played an important role in the changes observed in V1. Slower pieces with more similar scores between the two sections could have led to different outcomes. Similarly, passages with less interruption could impact intrapersonal strategies differently. On the contrary, it would be interesting in future studies to explicitly assess the role of body movement in inter- and intra-personal coordination processes during moments where performers do not play yet need to track the subtle timing of their peers to re-enter the piece accurately. Furthermore, we could question the generalizability of the results regarding not only the choice of the piece, but also the choice of the instrument and the musical genre. However, the reports of Berger (1997) on the shifts of focus between different metrical timescales in heavy-metal drumming as a support of different perceptual and motor coordination goals resonate deeply with our results and interpretation.

Third, our trade-off between a complex ecological context and an empirical format constitutes a limit to the generalization of the results, since the phenomena of interest have emerged from a non-habitual situation. However, the fact that a single modification in interpersonal coupling suffices to induce important shifts in intrapersonal coordination is revealing. While the perturbation method literally “sat” participants in unusual conditions, it highlighted how body movements can be used to cope with and make sense of a variety of uncertain situations (Dotov and Chemero, 2014). Such perturbation methods thus allow us to reveal the relevant functional variables that can be exploited and the patterns that can be spontaneously reorganized during interactions, especially when facing changes in environmental constraints (Glowinski et al., 2016). Therefore, the sort of generalization this kind of work permits is precisely the vicariousness and the situatedness of complex motor coordination. Furthermore, our interpretation of the present pattern of results encourages further investigation that could take the form of confirmatory studies. In particular, one could test if head motion at multiple scales frame auditory perception to support the coupling with instrumental action in properly ecological contexts (i.e., pieces performed in usual conditions only). Passages with differentiated demands in terms of timing control or dependance on other sections could be contrasted to verify if the patterns of head motion and interpersonal coordination change accordingly. Otherwise, experimental procedures where perturbations (e.g., unanticipated tempo changes and altered perception of peers) are applied only momentary could be used. This would allow to verify if players cope with the perturbation with similar changes in intrapersonal coordination and head motion as we observed here. In such contexts, longer excerpts could be used to enhance the ecological validity of the studies.

Another limitation of this study is the restricted focus on the analysis of movement (although musical information was taken into account to segment the data). Future studies should take musical information more closely into account. Separate audio recordings of the players would allow to investigate the potential acoustic correlates of the changes observed in motor behaviors, as they might affect the pressure of the bow for instance, and have consequences on the expressivity, the intensity and timbral features such as the harshness of the sonic outputs (Rozé et al., 2020). Similarly, more markers could be used for the motion capture. This would particularly help to parse postural fluctuations and genuine head motion. In the current study, we restricted the investigation of frequency composition to a range that corresponded to what was observable in bow motion and fitted the size of our windows of analysis. Yet periodicities at lower frequencies could probably be observed in head motion, in particular as consequences of torso movements. This would allow us to expand our understanding of intrapersonal coordination at multiple timescales and its relationship with interpersonal coupling.

Finally, future studies should integrate first-person inquiries about the strategies skilled experts use to cope with changes in complex and ecological situations. This would enable investigating whether changes in intrapersonal coupling and in head motion more particularly emerged from the coupling situation and from a tacit bodily know-how, or whether they rather reflect conscious, explicit strategies.

## Data availability statement

The raw data supporting the conclusions of this article will be made available by the authors, without undue reservation, to any qualified researcher.

## Ethics statement

The studies involving human participants were reviewed and approved by SIEMPRE Project Management Committee. The patients/participants provided their written informed consent to participate in this study.

## Author contributions

GV, AC, LF, and AD’A designed the study and collected the data. JL analyzed the results with the support of AT and AD’A. JL wrote the first draft of the manuscript. AT, GV, AC, LF, and AD’A contributed to the final manuscript. All authors contributed to the article and approved the submitted version.

## Footnotes

1
http://www.siempre.infomus.org

2 Although interpersonal coupling changed for V2 as well, the perturbation was more intense for V1, who completely lost sight of the crucial timing cues of the conductor in the process and gained an unprecedented visual access to V2’s motion. On the contrary, V2 already had access to V1 head and bow motion in the normal condition, although from a reverse angle. Moreover, V2’s score was sparser: they alternated short sequences where they played with sequences where they paused, making the assessment of their intrapersonal coordination non-suitable.
