# Supplementary material for: Interpersonal sensorimotor communication shapes intrapersonal coordination in a musical ensemble
Source: Front Hum Neurosci. 2022 Sep 29;16:899676. doi: 10.3389/fnhum.2022.899676 (PMC9556642; doi:10.3389/fnhum.2022.899676)
Supplement: Supplementary file 1 [file Data_Sheet_1.docx]

**SUPPLEMENTARY MATERIALS**

**Musical timing across conductors**

The average length of the excerpt was slightly shorter for the first conductor (56.7 +- .9 s) than the second conductor (59 +- .9 s). Using a Friedman test with the 2 conditions as the blocked factor to test the effect of the conductor, we found a significant difference (chi2 = 7.71; p = 0.0055). Relatedly, the inter-beat interval duration was shorter for the first conductor (258 +- 4 ms) than the second conductor (268 +- 4 ms) and the difference was significant (chi2 = 7.71; p = 0.0055). Finally, the coefficient of variation of the inter-beat interval duration was higher for the second conductor (0.068 +- 0.011) than for the first conductor (0.056 +- 0.008). This difference was only marginally significant (chi2 = 3.43; p = 0.0641). In short, the second conductor was leading the piece with a slightly slower pace and slightly more variability than the first conductor, reflecting the freedom of interpretation that was offered to them. However, the differences are very small in magnitude and should probably not impact other measures.

**Kinematic variables across conductors**

For each kinematic variable, we used Friedman tests to compare the results obtained between the 2 conductors, for each experimental condition separately. There were therefore 2 conductors to compare as column effects, 4 blocks (performers) as row effects to control, with 3 trials each. The results are reported in the tables below (tables S1 to S11). Most comparisons are non-significant. When a difference between conductors was observed for a variable (and only then), we performed a Friedman test to compare experimental conditions separately for each conductor. To interpret the results, we also computed the power spectral density of the motion of the baton and the head for each conductor (figure S1).

No difference between conductors was observed for any of the indices we derived from windowed cross-correlations, motion amplitude and spatial dispersion, power spectral density analysis (see tables S1-S9) and relative phase mean absolute angles (see table S14). Only a few differences were observed when analyzing phase relationships between head and bow at different metrical levels. Most of these differences were observed for the stability of the relative phase between head and bow motion (as indexed by vector length, see table S10). For each of these differences, we simultaneously report differences between experimental conditions for each conductor separately (see table S11). Some differences were also observed for relative phase mean angles: we present them in a similar fashion (tables S12-S13).


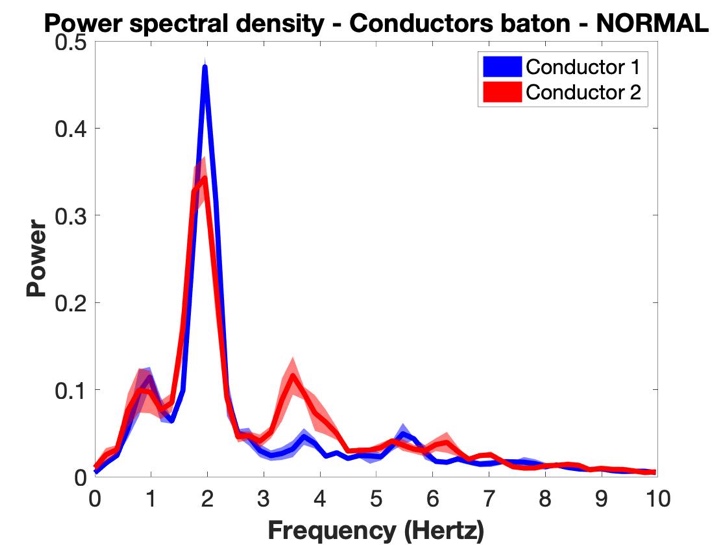

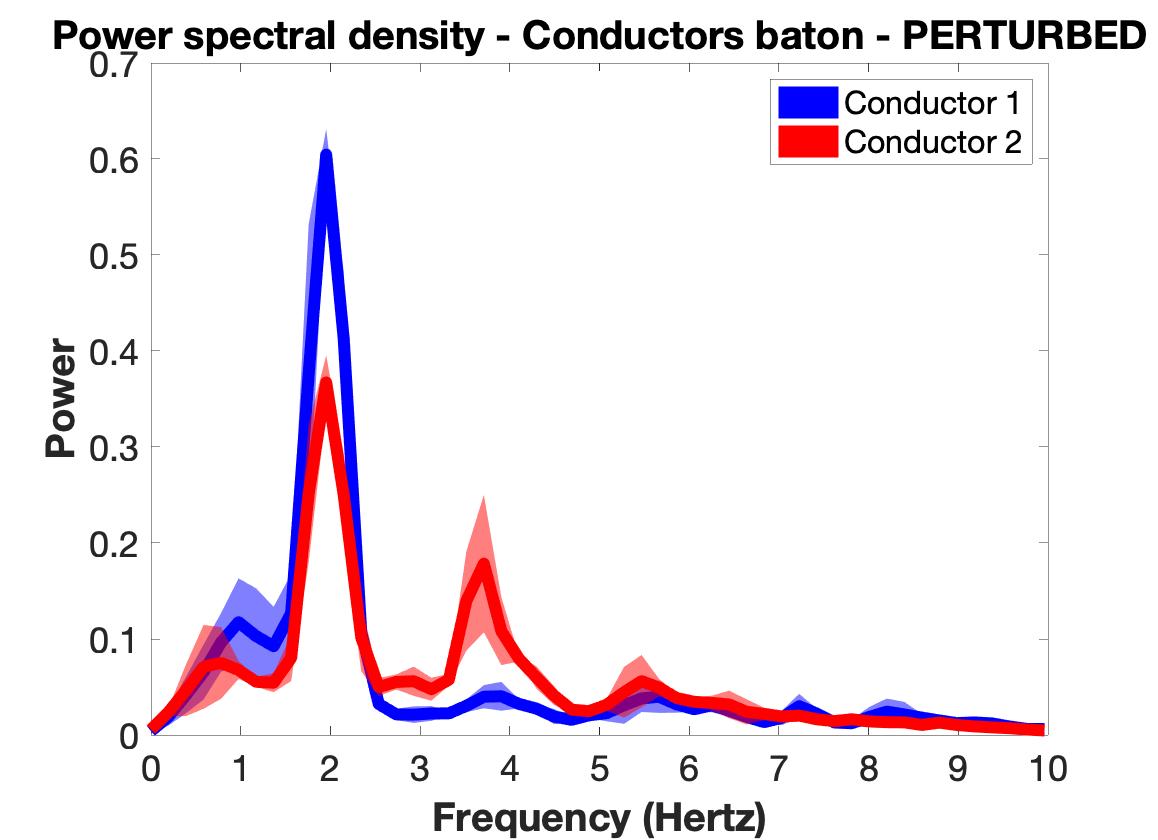


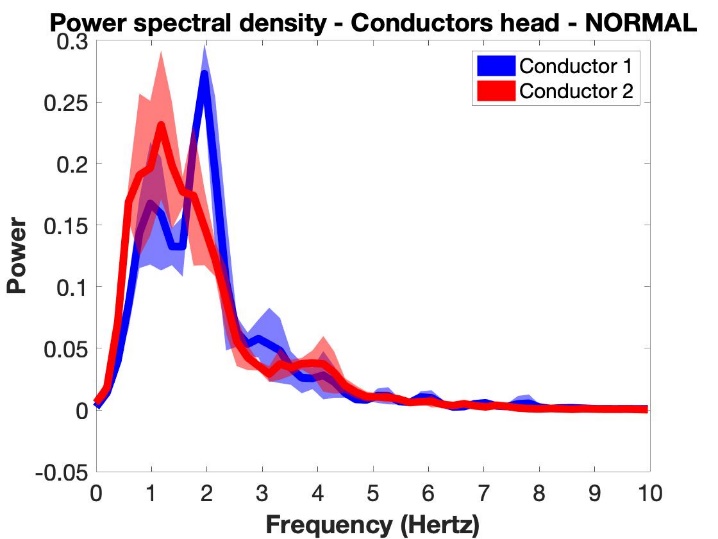

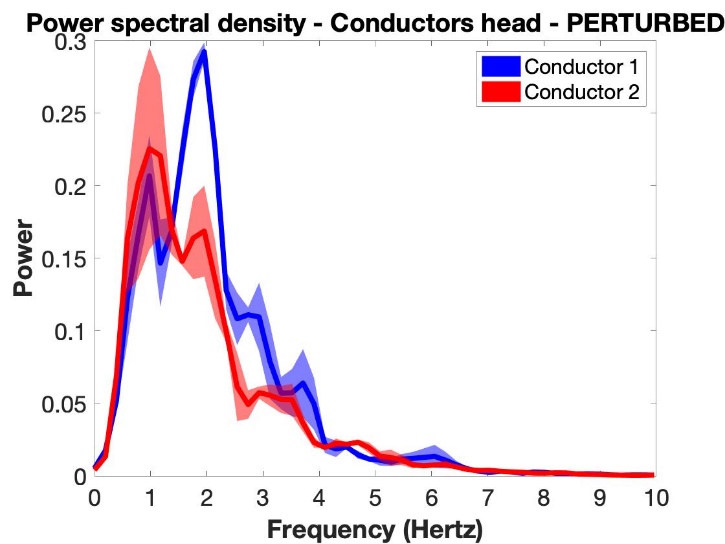


**Figure S1**. Power spectral density of the motion of the baton (upper figures) and the head (lower figures) in NORM (left figures) and PERT (right figures) conditions for each conductor. Whereas the overall profiles are similarly shaped across conductors, some differences were visible. Baton mostly moved around P2, as well as less prominently around P1 and P3. Major differences were observed around P2 which was more emphasized in the movements of conductor 1 than those of conductor 2, and the opposite pattern could be observed around P3. In PERT, there was also more motion from conductor 1 around P1. Similar frequency bands were found in conductors’ head motion, albeit with a different distribution and less sharp peaks. Activity around P2 still dominated the spectra, and that component was here again more emphasized by conductor 1 than conductor 2. On the contrary, in NORM at least, the component around P1 was more emphasized by conductor 2.

**Differences in relative phase stability across conductors**

At the level of the bar (P1), relative phase stability was significantly higher for conductor 2 (0.4883 +-0.0394) than for conductor 1 (0.4578 +-0.0391; chi2 = 4.7619, p = 0.0291) in NORM (see table S10). In PERT Relative phase stability was also higher for conductor 2 (0.4883 +-0.0394) than for conductor 1 (0.5330 +- 0.0443) but statistically this difference was only a marginal trend (chi2 = 3.0476; p = 0.0809). Looking at differences between conditions for each conductor, we observed higher relative phase stability in PERT for both conductors. For conductor 2, the difference was significant (NORM: 0.4578 +- 0.0391, PERT: 0.4883 +- 0.0394; chi2 = 4.7619, p = 0.0291). For conductor 1, there was a marginal trend (NORM: 0.5008 +- 0.0473, PERT: 0.5330 +- 0.0443; chi2 = 3.0476, p = 0.0809, see table S11). Overall, this reflects the results obtained by aggregating conductors, although the effect seemed to be more clearly marked for conductor 2.

At the level of the beat (P3), but only in PERT, stability was again significantly higher for conductor 2 (0.5477 +- 0.0208) than for conductor 1 (0.4933 +- 0.0373; chi2 = 4.7619, p = 0.0291; see table S10). We then observed, for conductor 2 but not for conductor 1, significantly higher relative phase stability in PERT (0.5477, +- 0.0208) than in NORM (0.4751 +- 0.0378, chi2 = 5.7619, p = 0.0164, see table S11). The increase in stability observed at P3 when aggregating conductors was thus only explained by the results obtained when conductor 2 was leading the orchestra.

At the level of half-notes (P2), and in PERT only, there was a marginal trend for relative phase stability to be this time lower with conductor 2 (0.5040 +- 0.0239) than with conductor 1 (0.5314 +- 0.0629; chi2 = 3.0476, p = 0.0809, see table S10). Examining the differences across conditions, for conductor 1 only, there was a marginal trend for relative phase stability to be higher in PERT (0.5314 +- 0.0629) than in NORM (0.4910 +- 0.0581; chi2 = 3.0476, p = 0.0809; see table S11).

Interestingly, the frequency component at which relative phase stability was higher for conductor 1 than for conductor 2 was P2, which was actually more emphasized by conductor 1 in both head and baton motion (see figure S1). Such an emphasis might have drawn violinists’ attention toward this metrical level, explaining the enhanced stability of their own head-bow coordination at this frequency, including in PERT where players could have internalized the conductor’s specific bodily articulated metrical framework. Similarly, the frequency components at which relative phase stability was higher for conductor 2 than for conductor 1 were P1 and P3. At P1, the difference was most pronounced in NORM, in which head motion of conductor 2 was more pronounced at this particular frequency bin. At P3, the baton motion of the conductor 2 was also more pronounced than those of conductor 1. These observations call again for an explanation based on attention, but the speculative nature of this interpretation calls for caution and more work that is beyond the scope of the present paper.

**Differences in relative phase mean angle across conductors**

At the level of half-notes (P2), relative phase mean angle values were slightly negative for conductor 2 (indicating that head motion preceded bow motion) and slightly positive for conductor 1 (indicating an opposite pattern). However, we only observed a marginal trend in PERT (conductor 1: 0.2151 +- 0.8709, conductor 2: -0.2528 +- 1.0568; chi2 = 3.0476; p = 0.0809, see table S12) and no difference was observed between conditions when analyzing trials performed with different conductors separately (see table S13), reflecting the results obtained when we aggregated them.

At the level of the beat (P3), a similar pattern was observed (positive mean angle values for conductor 1 and negative ones for conductor 2). This time the difference was significant but only in PERT (conductor 1: 0.0508 +- 0.4809, conductor 2: -0.4429 +- 0.5587; chi2 = 4.7619; p = 0.0291, see table S12). No difference was observed between conditions when analyzing trials performed with different conductors separately (see table S13), reflecting the results obtained when we aggregated them.

Differences in relative phase mean angle values at P2 and P3 are more difficult to interpret, as they were only observed in PERT (i.e. in absence of the vision of the conductor) and were significant only at P3. Since this did not entrain any difference between conditions and as their interpretation is beyond the scope of this paper, we let these results uninterpreted.

**Windowed cross-correlations across conductors**

| CC at lag 0 | NORM mean (std) | PERT mean (std) |
| --- | --- | --- |
| Conductor 1 | 0.1259 (0.0533) | 0.2221 (0.0788) |
| Conductor 2 | 0.1175 (0.0571) | 0.2308 (0.0695) |
| Chi-2 | 0.7619 | 1.1905 |
| P-value | 0.3827 | 0.2752 |

**Table S1**. Mean lag-0 cross-correlation coefficients between head and bow motion, compared across conductors for each experimental condition. No significant difference between conductors was observed.

| Peak CC | NORM mean (std) | PERT mean (std) |
| --- | --- | --- |
| Conductor 1 | 0.3547 (0.0489) | 0.4136 (0.0745) |
| Conductor 2 | 0.3595 (0.0575) | 0.4274 (0.0756) |
| Chi-2 | 0.0 | 1.1905 |
| P-value | 1.0 | 0.2752 |

**Table S2**. Mean peak cross-correlation coefficients between head and bow motion, compared across conductors for each experimental condition. No significant difference between conductors was observed.

| Lags of peak CC | NORM mean (std) | PERT mean (std) |
| --- | --- | --- |
| Conductor 1 | -1.250 (9.178) | -6.389 (12.624) |
| Conductor 2 | -5.139 (11.936) | -7.778 (8.401) |
| Chi-2 | 0.6049 | 0.1079 |
| P-value | 0.4367 | 0.7425 |

**Table S3**. Mean signed lag (in ms) at which peak cross-correlation coefficients between head and bow were observed, compared across conductors for each experimental condition. No significant difference between conductors was observed.

| Absolute lags of peak CC | NORM mean (std) | PERT mean (std) |
| --- | --- | --- |
| Conductor 1 | 38.472 (10.367) | 31.944 (14.102) |
| Conductor 2 | 39.306 (8.682) | 33.889 (8.761) |
| Chi-2 | 0.1079 | 0.5875 |
| P-value | 0.7425 | 0.4434 |

**Table S4**. Mean absolute lag (in ms) at which peak cross-correlation coefficients between head and bow were observed, compared across conductors for each experimental condition. No significant difference between conductors was observed.

| CC lags std | NORM mean (std) | PERT mean (std) |
| --- | --- | --- |
| Conductor 1 | 42.399 (9.338) | 32.639 (14.780) |
| Conductor 2 | 41.711 (10.132) | 32.639 (9.637) |
| Chi-2 | 0.0476 | 0.4286 |
| P-value | 0.8273 | 0.5127 |

**Table S5**. Standard deviation of the lags at which peak cross-correlation coefficients between head and bow were observed, compared across conductors for each experimental condition. No significant difference between conductors was observed.

**Motion amplitude and spatial dispersion across conductors**

| Head Convex Hull | NORM mean (std) | PERT mean (std) |
| --- | --- | --- |
| Conductor 1 | 50.027 (61.039) | 10.877 (9.510) |
| Conductor 2 | 41.426 (47.063) | 7.731 (8.960) |
| Chi-2 | 0.4286 | 1.7143 |
| P-value | 0.5127 | 0.1904 |

| Head Mean  Inter-Distance | NORM mean (std) | PERT mean (std) |
| --- | --- | --- |
| Conductor 1 | 43.024 (25.556) | 26.148 (10.031) |
| Conductor 2 | 42.975 (22.905) | 25.081 (11.795) |
| Chi-2 | 0.1905 | 0.7619 |
| P-value | 0.6625 | 0.3827 |

**Table S6**. Convex hull volume (in dm3) and mean inter-distance (in mm) between positional datapoint of the head motion, compared across conductors for each experimental condition. No significant difference between conductors was observed.

| Bow Convex Hull | NORM mean (std) | PERT mean (std) |
| --- | --- | --- |
| Conductor 1 | 1.1304 (0.7734) | 0.9630 (0.2626) |
| Conductor 2 | 1.1905 (0.7581) | 0.8610 (0.434) |
| Chi-2 | 0.4286 | 0.0 |
| P-value | 0.5127 | 1.0 |

| Bow mean  inter-distance | NORM mean (std) | PERT mean (std) |
| --- | --- | --- |
| Conductor 1 | 99.839 (17.374) | 94.696 (4.020) |
| Conductor 2 | 103.881 (14.951) | 98.346 (4.452) |
| Chi-2 | 3.048 | 1.714 |
| P-value | 0.081 | 0.1904 |

**Table S7**. Convex hull (a) and mean inter-distance between positional datapoint (b) of the head motion, compared across conductors for each experimental condition. No significant difference between conductors was observed.

**Power spectral density across conductors**

| Head PSD - P1 | NORM mean (std) | PERT mean (std) |
| --- | --- | --- |
| Conductor 1 | 0.1819 (0.0415) | 0.1531 (0.0535) |
| Conductor 2 | 0.1640 (0.0359) | 0.1538 (0.0502) |
| Chi-2 | 2.3333 | 0.0476 |
| P-value | 0.1266 | 0.8273 |

| Head PSD – P2 | NORM mean (std) | PERT mean (std) |
| --- | --- | --- |
| Conductor 1 | 0.0880 (0.0303) | 0.1135 (0.0386) |
| Conductor 2 | 0.1034 (0.0702) | 0.1205 (0.0278) |
| Chi-2 | 0.4286 | 1.7143 |
| P-value | 0.5127 | 0.1904 |

| Head PSD – P3 | NORM mean (std) | PERT mean (std) |
| --- | --- | --- |
| Conductor 1 | 0.0355 (0.0223) | 0.1135 (0.0386) |
| Conductor 2 | 0.0309 (0.0127) | 0.1205 (0.0278) |
| Chi-2 | 0.1905 | 1.7143 |
| P-value | 0.6625 | 0.1904 |

| Head PSD – P4 | NORM mean (std) | PERT mean (std) |
| --- | --- | --- |
| Conductor 1 | 0.0064 (0.0095) | 0.0139 (0.0182) |
| Conductor 2 | 0.0080 (0.0108) | 0.0132 (0.0175) |
| Chi-2 | 1.7143 | 0.4286 |
| P-value | 0.1904 | 0.5127 |

**Table S8**. Power values (in db/Hz) extracted from the power spectral density spectra at the 4 frequency bins that corresponded to musical metrical levels, compared across conductors for each experimental condition for the motion of the head. No significant difference between conductors was observed.

| Bow PSD - P1 | NORM mean (std) | PERT mean (std) |
| --- | --- | --- |
| Conductor 1 | 0.1173 (0.0299) | 0.1274 (0.0506) |
| Conductor 2 | 0.1320 (0.0388) | 0.1391 (0.0409) |
| Chi-2 | 1.1905 | 1.7143 |
| P-value | 0.2752 | 0.1904 |

| Bow PSD – P2 | NORM mean (std) | PERT mean (std) |
| --- | --- | --- |
| Conductor 1 | 0.1037 (0.0280) | 0.0987 (0.0115) |
| Conductor 2 | 0.1146 (0.0298) | 0.1168 (0.0177) |
| Chi-2 | 0.0476 | 2.3333 |
| P-value | 0.8273 | 0.1266 |

| Bow PSD – P3 | NORM mean (std) | PERT mean (std) |
| --- | --- | --- |
| Conductor 1 | 0.1519 (0.0754) | 0.1515 (0.0659) |
| Conductor 2 | 0.1514 (0.0554) | 0.1415 (0.0713) |
| Chi-2 | 0.0 | 0.1905 |
| P-value | 1.0 | 0.6625 |

| Bow PSD – P4 | NORM mean (std) | PERT mean (std) |
| --- | --- | --- |
| Conductor 1 | 0.0753 (0.0293) | 0.1007 (0.0365) |
| Conductor 2 | 0.0821 (0.0258) | 0.0878 (0.0144) |
| Chi-2 | 0.1905 | 0.4286 |
| P-value | 0.6625 | 0.5127 |

**Table S9**. Power values (in db/Hz) extracted from the power spectral density spectra at the 4 frequency bins that corresponded to musical metrical levels, compared across conductors for each experimental condition for the motion of the head. No significant difference between conductors was observed.

**Relative phase analysis** **across conductors**

| RP Vector length  P1 | NORM mean (std) | PERT mean (std) |
| --- | --- | --- |
| Conductor 1 | 0.4578 (0.0391) | 0.5008 (0.0473) |
| Conductor 2 | 0.4883 (0.0394) | 0.5330 (0.0443) |
| Chi-2 | 4.7619 | 3.0476 |
| P-value | **0.0291** | **0.0809** |

| RP Vector length  P2 | NORM mean (std) | PERT mean (std) |
| --- | --- | --- |
| Conductor 1 | 0.4910 (0.0581) | 0.5314 (0.0629) |
| Conductor 2 | 0.4954 (0.0684) | 0.5040 (0.0239) |
| Chi-2 | 0.0476 | 3.0476 |
| P-value | 0.8273 | **0.0809** |

| RP Vector length  P3 | NORM mean (std) | PERT mean (std) |
| --- | --- | --- |
| Conductor 1 | 0.4880 (0.0399) | 0.4933 (0.0373) |
| Conductor 2 | 0.4751 (0.0378) | 0.5477 (0.0208) |
| Chi-2 | 0.0476 | 4.7619 |
| P-value | 0.8273 | **0.0291** |

| RP Vector length  P4 | NORM mean (std) | PERT mean (std) |
| --- | --- | --- |
| Conductor 1 | 0.4984 (0.1065) | 0.5240 (0.0930) |
| Conductor 2 | 0.5292 (0.0600) | 0.5505 (0.0972) |
| Chi-2 | 0.4286 | 0.7619 |
| P-value | 0.5127 | 0.3827 |

**Table S10**. Vector length of the relative phase between head and bow motion across conductors, for each experimental condition and at each relevant metrical level. At P1, trials with conductor 2 led to greater relative phase stability between head and bow. The difference was significant in NORM, and a marginal trend was observed in PERT. At P2, there was a marginal trend indicating that, this time, relative phase stability was slightly lower for conductor 2 in PERT. At P3, relative phase stability was higher for conductor 2 in PERT. Other comparisons were not significant.

| RP Vector length  P1 | Conductor 1  mean (std) | Conductor 2  mean (std) |
| --- | --- | --- |
| NORM | 0.5008 (0.0473) | 0.4578 (0.0391) |
| PERT | 0.5330 (0.0443) | 0.4883 (0.0394) |
| Chi-2 | 3.0476 | 4.7619 |
| P-value | **0.0809** | **0.0291** |

| RP Vector length  P2 | Conductor 1  mean (std) | Conductor 2  mean (std) |
| --- | --- | --- |
| NORM | 0.4910 (0.0581) | 0.4954 (0.0684) |
| PERT | 0.5314 (0.0629) | 0.5040 (0.0239) |
| Chi-2 | 3.0476 | 0.0476 |
| P-value | **0.0809** | 0.8273 |

| RP Vector length  P3 | Conductor 1  mean (std) | Conductor 2  mean (std) |
| --- | --- | --- |
| NORM | 0.4880 (0.0399) | 0.4751 (0.0378) |
| PERT | 0.4933 (0.0373) | 0.5477 (0.0208) |
| Chi-2 | 0.4286 | 5.7619 |
| P-value | 0.5127 | **0.0164** |

**Table S11**. Vector length of the relative phase between head and bow motion across experimental conditions, for each conductor, at each metrical level where differences between conductors were observed. At P1, relative phase stability was higher in PERT than in NORM. The difference was significant for conductor 2 and there was a marginal trend for conductor 1 as well. Overall, this reflects the results observed in the global comparisons, which did not differentiate between conductors. At P2, there was a marginal trend for relative phase stability to be higher in PERT for conductor 1 (but no difference for conductor 2). At P3, relative phase stability was significantly higher in PERT for conductor 2 (but no difference was observed for conductor 1).

| RP Mean Angle  P1 | NORM  mean (std) | PERT  mean (std) |
| --- | --- | --- |
| Conductor 1 | 0.0879 (0.5167) | -0.1939 (0.3929) |
| Conductor 2 | -0.0114 (0.0874) | -0.0163 (0.3695) |
| Chi-2 | 0.1905 | 0.4286 |
| P-value | 0.6625 | 0.5127 |

| RP Mean Angle  P2 | NORM  mean (std) | PERT  mean (std) |
| --- | --- | --- |
| Conductor 1 | 0.2030 (0.9874) | 0.2151 (0.8709) |
| Conductor 2 | -0.0931 (1.3166) | -0.2528 (1.0568) |
| Chi-2 | 0.1905 | 3.0476 |
| P-value | 0.6625 | **0.0809** |

| RP Mean Angle  P3 | NORM  mean (std) | PERT  mean (std) |
| --- | --- | --- |
| Conductor 1 | 0.2519 (0.8897) | 0.0508 (0.4809) |
| Conductor 2 | -0.3470 (0.6138) | -0.4429 (0.5587) |
| Chi-2 | 0.4286 | 4.7619 |
| P-value | 0.5127 | **0.0291** |

| RP Mean Angle  P4 | NORM  mean (std) | PERT  mean (std) |
| --- | --- | --- |
| Conductor 1 | -0.4664 (0.6761) | -0.0536 (1.4428) |
| Conductor 2 | -0.8984 (0.9892) | -0.4714 (0.6545) |
| Chi-2 | 0.0476 | 0.4286 |
| P-value | 0.8273 | 0.5127 |

**Table S12**. Mean relative angles (in radians) of the relative phase between head and bow motion across conductors, for each experimental condition and at each relevant metrical level. At P2, conductor 1 led to positive mean angle values and conductor 2 led to negative mean angle, but the difference only presented a marginal trend in PERT. At P3, a similar pattern was observed: conductor 2 led to negative mean angle values and conductor 1 to positive ones. This difference was significant only in PERT. Other comparisons were not significant.

| RP Mean Angle  P2 | Conductor 1  mean (std) | Conductor 2  mean (std) |
| --- | --- | --- |
| NORM | 0.2030 (0.9874) | -0.0931 (1.3166) |
| PERT | 0.2151 (0.8709) | -0.2528 (1.0568) |
| Chi-2 | 2.3333 | 1.1905 |
| P-value | 0.1266 | 0.2752 |

| RP Mean Angle  P3 | Conductor 1  mean (std) | Conductor 2  mean (std) |
| --- | --- | --- |
| NORM | 0.2519 (0.8897) | -0.3470 (0.6138) |
| PERT | 0.0508 (0.4809) | -0.4429 (0.5587) |
| Chi-2 | 0.1905 | 0.1905 |
| P-value | 0.6625 | 0.6625 |

**Table S13**. Mean relative angles (in radians) of the relative phase between head and bow motion across experimental conditions, for each conductor and at the metrical levels where differences between conductors were observed. There was no difference between condition for any of the conductors. This reflects the results observed in the global comparisons, which did not differentiate between conductors.

| RP - P1  Mean Absolute Angle | NORM  mean (std) | PERT  mean (std) |
| --- | --- | --- |
| Conductor 1 | 1.2293 (0.1187) | 1.1057 (0.1047) |
| Conductor 2 | 1.1687 (0.0499) | 1.1399 (0.1455) |
| Chi-2 | 0.4286 | 0.4286 |
| P-value | 0.5127 | 0.5127 |

| **RP - P2**  **Mean Absolute Angle** | NORM  mean (std) | PERT  mean (std) |
| --- | --- | --- |
| Conductor 1 | 1.4015 (0.1414) | 1.2813 (0.1589) |
| Conductor 2 | 1.4194 (0.1563) | 1.3196 (0.1925) |
| Chi-2 | 0.4286 | 0.7619 |
| P-value | 0.5127 | 0.3827 |

| RP - P3  Mean Absolute Angle | NORM  mean (std) | PERT  mean (std) |
| --- | --- | --- |
| Conductor 1 | 1.4261 (0.1460) | 1.2454 (0.1628) |
| Conductor 2 | 1.3841 (0.0740) | 1.2460 (0.0818) |
| Chi-2 | 0.7619 | 0.0000 |
| P-value | 0.3827 | 1.000 |

| RP - P4  Mean Absolute Angle | NORM  mean (std) | PERT  mean (std) |
| --- | --- | --- |
| Conductor 1 | 1.5149 (0.1064) | 1.4129 (0.2469) |
| Conductor 2 | 1.5623 (0.1200) | 1.2918 (0.1255) |
| Chi-2 | 0.0476 | 2.333 |
| P-value | 0.8273 | 0.1266 |

**Table S14**. Mean absolute angles (in radians) of the relative phase between head and bow motion across conductors, for each experimental condition and at each relevant metrical level. No significant difference was observed between conductors.
